# Erbin exerts a protective effect against inflammatory bowel disease by suppressing autophagic cell death

**DOI:** 10.18632/oncotarget.23925

**Published:** 2018-01-04

**Authors:** Tong Shen, Shi Li, Ling-Dong Cai, Jing-Lin Liu, Chu-Yi Wang, Wen-Juan Gan, Xiu-Ming Li, Jing-Ru Wang, Li-Na Sun, Min Deng, Yu-Hong Liu, Jian-Ming Li

**Affiliations:** ^1^ Department of Pathology, Soochow University Medical School, Suzhou 215123, People’s Republic of China; ^2^ Department of Pathology, Baoan Hospital, Southern Medical University, Shenzhen 518101, People’s Republic of China

**Keywords:** Erbin, inflammatory bowel disease, autophagy, cell death

## Abstract

The pathogenesis and key functional molecules involved in inflammatory bowel disease (IBD) including Crohn's disease (CD) and ulcerative colitis (UC) remain unclear. Here, we reported that Erbin, a protein required for the polarity of epithelial cells, is conserved across species and highly expressed in the intestinal mucosa in mice and zebrafish. Pathologically, Erbin expression in the intestinal mucosa was significantly decreased in DSS induced acute colitis mice, IL-10 deficient mice and clinical biopsy specimens from patients with ulcerative colitis. Moreover, Erbin deficient mice are more susceptible to experimental colitis, exhibiting more severe intestinal barrier disruption, with increased histological scores and excessive production of proinflammatory cytokines. Mechanistically, Erbin deficiency or knockdown significantly exacerbated activation of autophagic program and autophagic cell death *in vivo* and *in vitro*. And, inhibition of autophagy by Chloroquine attenuates excessive inflammatory response in the DSS-induced colitis mouse model of Erbin deletion. Generally, our study uncovers a crucial role of Erbin in autophagic cell death and IBD, giving rise to a new strategy for IBD therapy by inhibiting excessive activation of autophagy and autophagic cell death.

## INTRODUCTION

Inflammatory bowel disease (IBD) that includes both ulcerative colitis (UC) and Crohn’s disease (CD) [1], is a chronic inflammatory disease of the gastrointestinal tract [2]. The prompt activation of the intestinal immune system and an inflammatory reaction is essential for the host to maintain gut homeostasis. However, dysfunctional immune response in the gut mucosa characterized as infiltration of neutrophils and macrophages, excessive release of chemokines and cytokines, disruption of colonic epithelium, is usually associated with IBD. However, the mechanisms of IBD and key genes involved are still not well-characterized.

Erbin, a member of the leucine-rich repeat and PDZ domain (LAP) family, was originally identified as a binding protein to ErbB2 (also known as HER-2 or Neu) [3]. Increasing studies indicated that Erbin plays important roles in cell polarization, receptor localization, and signal transduction. In our previous study [4], Erbin interacts with EGFR and promotes tumourigenesis and tumour growth in CRC. Recently, we reported that Erbin interacts with ER-α and helps for Tamoxifen resistance in hepatocellular carcinoma [5]. Besides these functions, Erbin was suggested involved in Nod2-dependent activation of NF-κB and cytokine secretion by interacting with Nod2 [6]. Nevertheless, the role of Erbin in IBD is absolutely unknown.

Autophagy is a fundamental cellular process in eukaryotes ongoing under physiologic circumstances in almost all cell types of the human organism and upregulated by various stress conditions including those leading to inflammation. An array of pathogen-and damage-associated molecular patterns (PAMPs) can induce or inhibit autophagy, which creates a set of feedback loops between autophagy and inflammation to mediate host defence and limit tissue damage [7]. In its most common form of autophagy, fusion of the autophagosome with endolysosomal vesicles leads to degradation and recycling of the sequestered substrates. Many of the substrates found within autophagosomes are those that threaten cell viability, such as damaged organelles, protein aggregates and intracellular pathogens [8]. Defects in autophagy may lead to an impaired antibacterial response and bacterial clearance, which contribute to persistent chronic inflammation and IBD pathogenesis. Traditionally, down-regulation or defect of autophagy will contribute to IBD pathogenesis. Comparing with that about the role of autophagy defect in IBD pathogenesis, our knowing about the excessive activation of autophagy program in IBD pathogenesis is still lack.

Here, we found that Erbin deficient mice are more susceptible to experimental colitis. Moreover, Erbin deficiency exacerbates activation of autophagic program in experiment colitis mouse model *in vivo* and knockdown of Erbin aggravates TNF-α inducing autophagic cell death *in vitro*. Thus, we uncover a key role of Erbin in autophagic cell death and IBD. Our data give rise to a potential new strategy for IBD therapy by inhibiting excessive activation of autophagy program and autophagic cell death.

## RESULTS

### Erbin is highly expressed in the intestinal mucosa of mice and zebrafish

**Figure 1:**
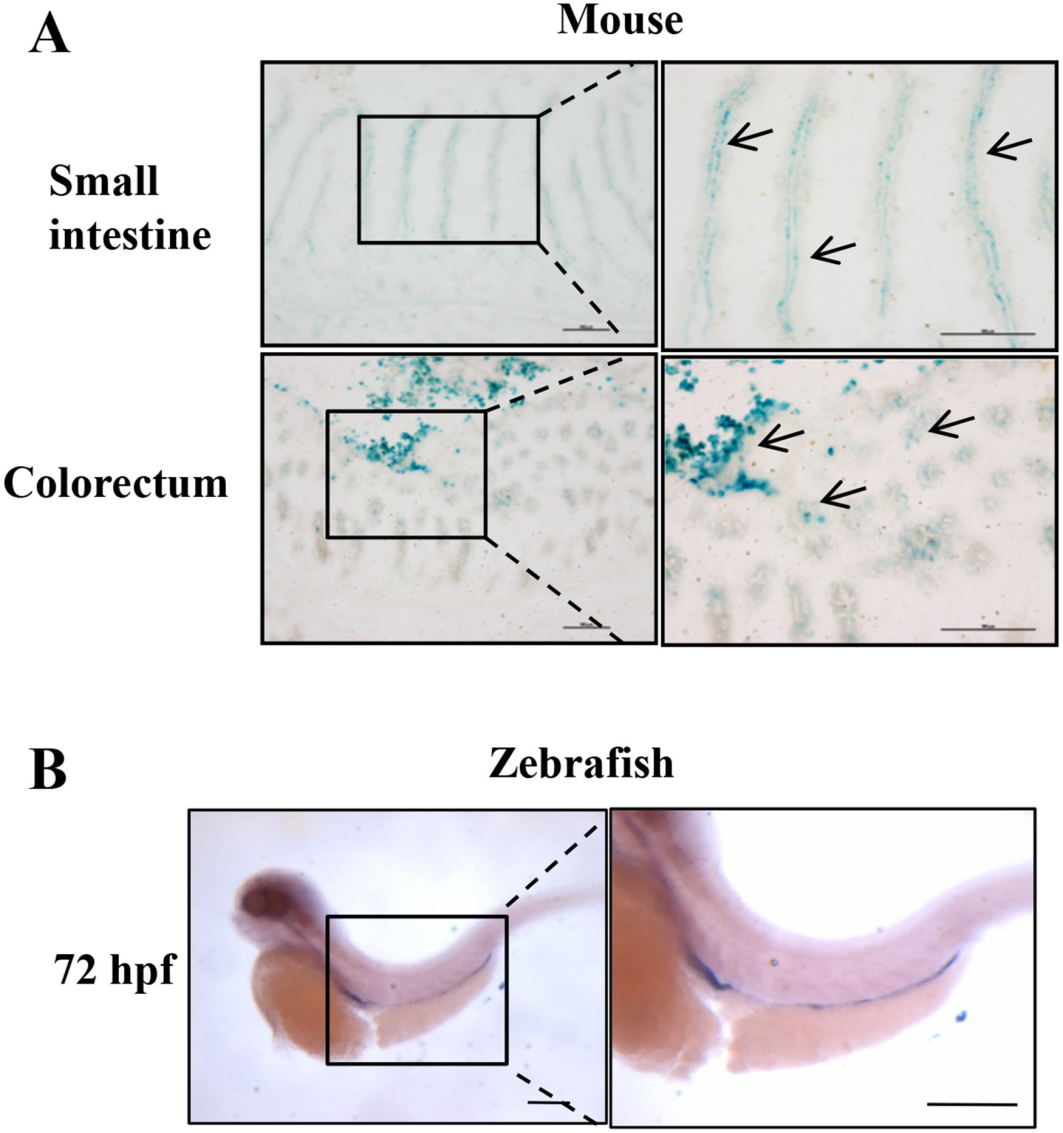
Intestinal specific expression of Erbin in mouse and zebrafish **(A)** X-gal–stained small intestine and colon using an Erbin reporter mouse. **(B)** Erbin expression in zebrafish by whole-mount *in situ* hybridization.

To investigate the expression pattern of Erbin in mice, we used a well-established Erbin reporter mice (donated by Mei lin) [9], in which expression of Erbin can be tracked with a beta-galactosidase enzyme, encoded by the lacZ gene. Interestingly, Erbin, as tracked with X-galactosidase (X-gal) staining (Figure 1A), was highly expressed in mucosa of small intestine and colorectum of mice.

Moreover, we investigated the expression pattern of Erbin in zebrafish, a model animal. The distribution of the transcript of Erbin during the developmental stages of zebrafish was studied by whole-mount *in situ* hybridization with digoxigenin-labeled RNA probes corresponding to full length Erbin cDNA. The transcripts of Erbin were detected from the zebrafish embryonic to 180 hours post fertilization (hpf). Erbin was expressed exclusively in the gut at 72 hpf (Figure 1B), while the foregut and hindgut had developed completely since 26 hpf [10].

### Erbin expression in the intestinal mucosa is significantly decreased in experiment colitis mouse model and patients with ulcerative colitis

Physiologically, Erbin is high expressed in the intestinal mucosa of mice and even zebrafish. Next, we study the expression pattern of Erbin in experiment colitis mouse model and patients with ulcerative colitis.

**Figure 2:**
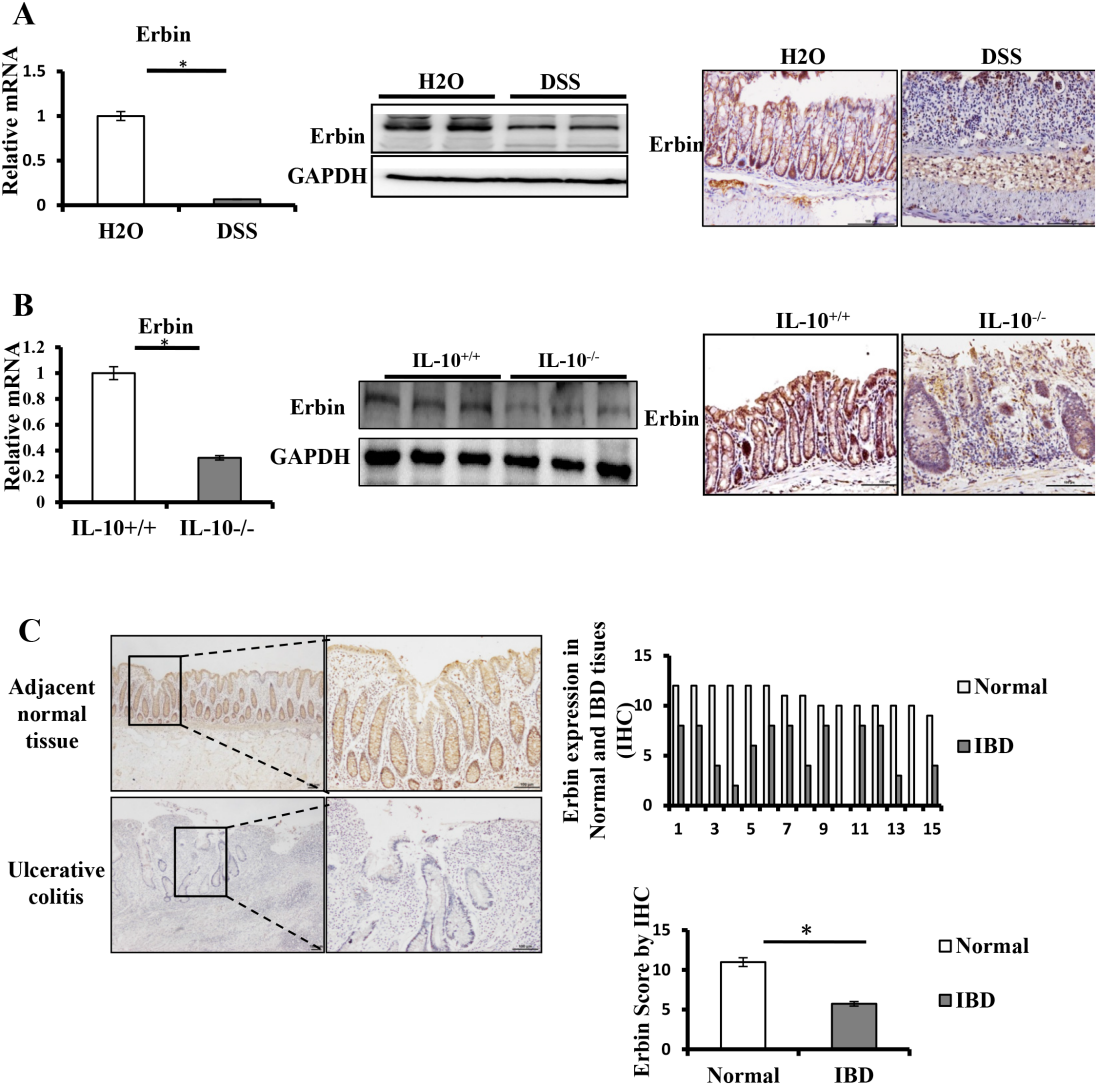
Expressions of Erbin in DSS-induced colitis model of mice and IL-10^-/-^ mice **(A)** Erbin expressions in colon of DSS-induced colitis model of mice by RT-qPCR, Western Blots (WB), and immunohistochemical staining (IHC). **(B)** Erbin expressions in colon of IL-10^-/-^ mice by RT-qPCR, WB and IHC. **(C)** Expression of Erbin in colon of 15 patients with ulcerative colitis by IHC. n =15 in each group; Scale bars = 50 μm (C).

Two experiment colitis mouse models were used in this study. In DSS-induced colitis mouse model, mice exhibited similar symptoms and histological changes to those observed in human IBD, particularly UC (Supplementary Figure 1A) [11]. In IL-10 knockout (IL-10^-/-^) mice, mice are growth retarded and anemic and suffering from chronic enterocolitis (Supplementary Figure 1B) [12]. Immunohistochemical staining showed that Erbin expression in the intestinal mucosa was significantly decreased in two experiment colitis mouse models. Moreover, Erbin was localized largely in the intestinal mucosa and throughout the entire crypt. In isolated colorectum from DSS-induced colitis mice and IL-10 knockout mice, Erbin expression at both transcriptional and translational levels was markedly decreased compared with that in those control mice (Figure 2A and 2B).

To evaluate expression of Erbin protein in patients with ulcerative colitis, 15 specimens for colorectal mucosa biopsy were used to stain with anti-Erbin antibodies. The diagnosis of ulcerative colitis had been previously made on the basis of repeated colonoscopy, histological tests, and contrast radiography. Compared with that in relatively normal colonic mucosa from control group, Erbin expression was significantly decreased in patients with ulcerative colitis (p <0.05) (Figure 2C).

### Erbin deficient mice are more susceptible to experimental colitis

To further investigate the role of Erbin in intestinal homeostasis, we established an Erbin knockout (Erbin^-/-^) mice, in which Erbin was deleted by a CRISPR/Pro System. The deletion in exon 16 of Erbin gene (Supplementary Figure 2A), which results in a frameshift and immediate termination codon, led to deletion of Erbin at both transcriptional and translational levels in gut of the transgenic mice Erbin^-/-^ (Supplementary Figure 2B).

**Figure 3:**
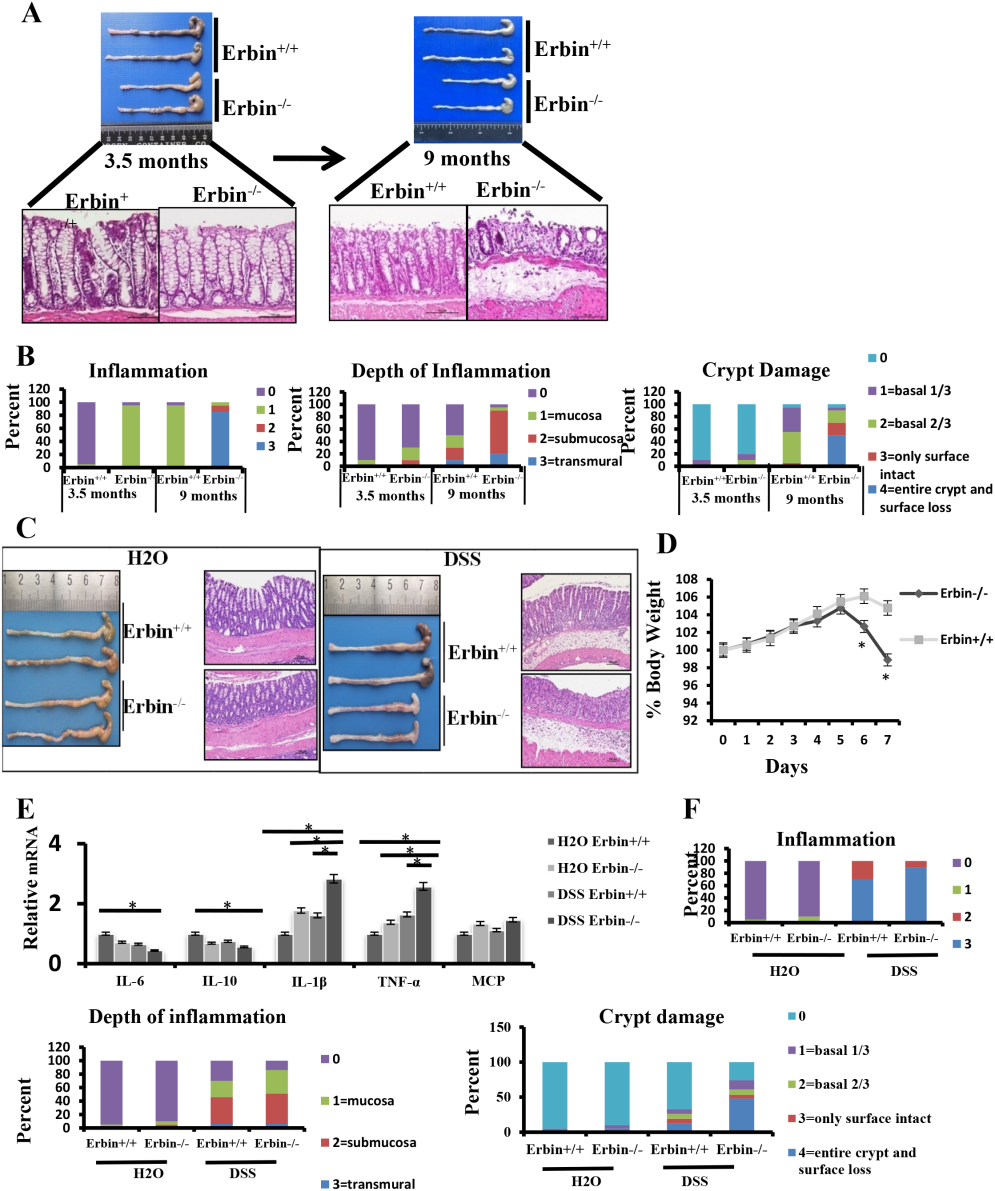
Intestinal inflammatory response and epithelial injury in experimental colitis mouse model after Erbin was deleted **(A)** Gross appearance and representative H&E staining of colorectum in 3.5-month-old and 9- month-old Erbin^-/-^ and wild type (WT) mice. **(B)** Histopathological scorings of the inflammation, depth of inflammation, and crypt damage of colorectum in 3.5-month-old and 9-month-old Erbin^-/-^ and WT mice. Data are represented as the percentage of mice per group with the indicated score. **(C)** Gross appearance and representative H&E staining of colorectum in Erbin^-/-^ and WT mice with or without DSS treatment. **(D)** Weight loss of Erbin^-/-^ and WT mice after DSS treatment. **(E)** Expression of IL-6, IL-10, IL-1β, TNF-α and MCP of colorectum in Erbin^-/-^and WT mice after DSS treatment. **(F)** Histopathological scorings of the inflammation, depth of inflammation, and crypt damage of colorectum in Erbin^-/-^ and WT mice with DSS treatment. ^*^P < 0.05 (t-test) (D and E). Data are expressed as means±SEM (E). Scale bars = 50 μm (A and C).

Interestingly, physiologically, in 9-month-old Erbin^-/-^ mice, colorectal epithelium and crypts were markedly damaged (Figure 3A) compared with that in control mice. Histopathological scorings for the inflammation, depth of inflammation and crypt damage indicated that more severe intestinal inflammatory response and epithelial injury were found in 9-month-old Erbin^-/-^ mice or even 3.5- month-old Erbin^-/-^ mice compared with that in control mice (Figure 3B).

**Figure 4:**
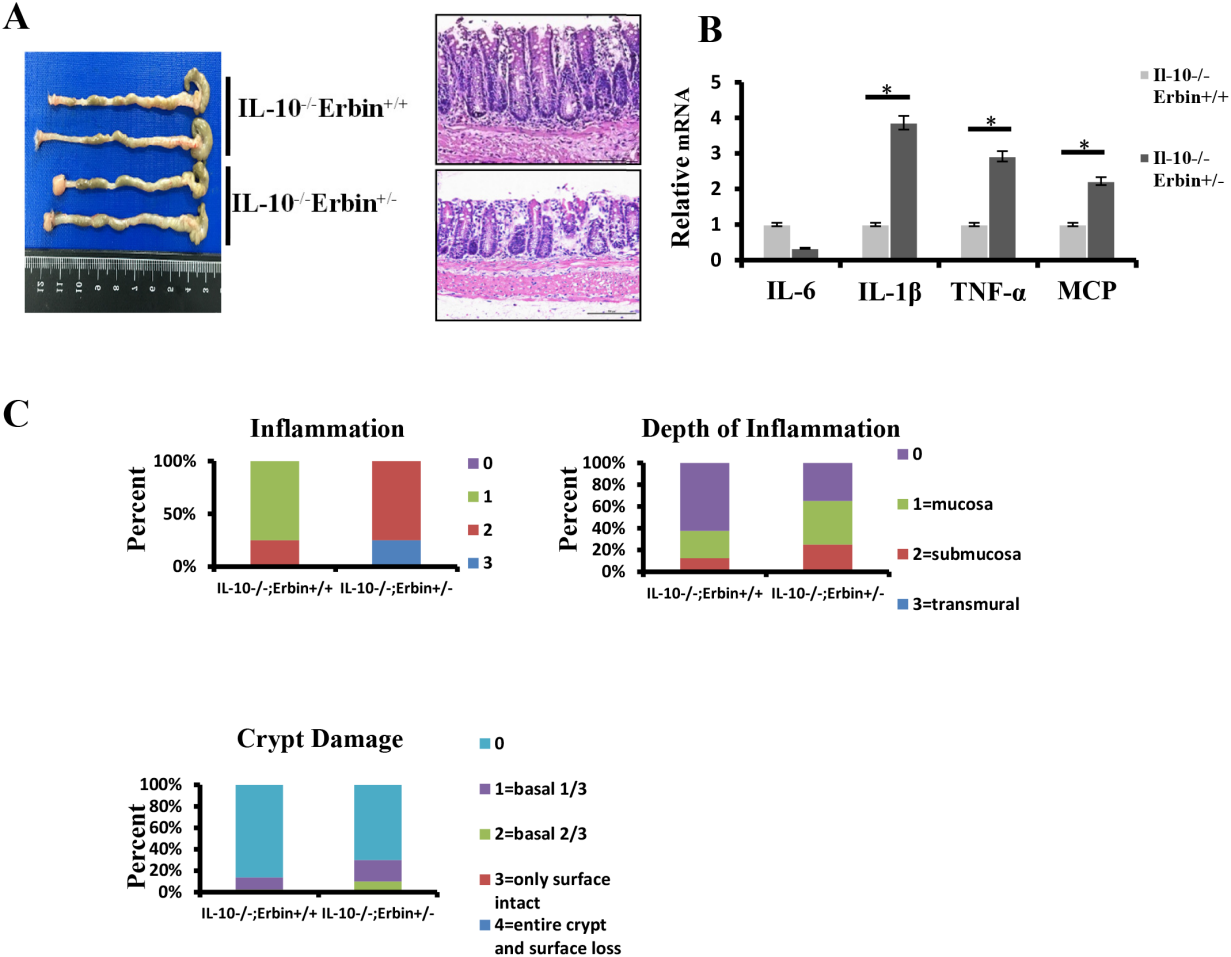
Intestinal inflammatory response and epithelial injury in IL-10^-/-^ Erbin^+/-^ mice **(A)** Gross appearance and representative H&E staining of colorectum in IL-10^-/-^Erbin^+/-^ and IL-10^-/-^Erbin^+/+^ mice. **(B)** Expression of cytokines IL-6, IL-10, IL-1β, TNF-α and MCP of colorectum from IL-10^-/-^Erbin^+/-^ and IL-10^-/-^Erbin^+/+^ mice. **(C)** Histopathological scoring of the inflammation, depth of inflammation, and crypt damage of IL-10^-/-^Erbin^+/-^ and IL-10^-/-^Erbin^+/+^ mice. Data are represented as the percentage of mice per group with the indicated score. ^*^P < 0.05 (t-test). Data are expressed as means±SEM (B). Scale bars = 50 μm (A).

Furthermore, we established a DSS induced experimental mouse model of colitis, in which mice were exposed to DSS (2.5% wt/vol) drink for 7 days. We found that Erbin^-/-^ mice are more susceptible to DSS exposure. Erbin^-/-^ mice exhibited more severe clinical symptoms such as loss of body weight, loose feces/watery diarrhea, and fecal blood than control mice (Figure 3C and 3D). Scorings for the inflammation, depth of inflammation and crypt damage demonstrated that Erbin^-/-^ mice had more severe intestinal inflammatory response and epithelial injury, indicated as enlargement of the mesenteric lymph nodes, decrease in size of the cecum, loss of nearly 50% crypts, infiltration of inflammatory cells, and massive edema in the submucosa, than control mice (Figure 3F). Expression profiles for cytokines in colon showed that mRNA expression levels of IL-1β, TNF-α and MCP were greatly increased and mRNA expression levels of IL-10 and IL-6 were significantly decreased in Erbin^-/-^ mice compared with that in control mice after DSS treatment (Figure 3E). We also generated IL-10^-/-^ Erbin^+/-^ mice (IL-10^-/-^Erbin^-/-^ could not be available in our study) to determine the role of Erbin in colitis. Compared to IL-10^-/-^ mice, IL-10^-/-^ Erbin^+/-^ mice exhibited more severe gut inflammation (Figure 4A and 4C), higher mRNA expression levels of IL-1β, TNF-α and MCP (Figure 4B).

Interestingly, when Paneth cell were stained in small intestine by phloxine-tartrazine staining, we found a decrease in the number of Paneth cell in the terminal ileum of the Erbin^-/-^ mice compared with that in control wild type (WT) mice, in both physiological condition and DSS treatment (Supplementary Figure 3A). qPCR analysis showed that mRNA expression levels of Lysozyme (marker for Paneth cells) and Cryptidin were significantly decreased in ileum of the Erbin^-/-^ mice compared with that in WT mice without DSS treatment. After DSS treatment, the expressions of Lysozyme and Cryptidin was dramatically decreased in Erbin^-/-^ mice compared with that in WT mice (Supplementary Figure 3B).

### Erbin deficiency exacerbates activation of autophagic program in experiment colitis mouse model *in vivo*

We found that Erbin deficiency led to aggravated inflammatory response and damage of gut. The key program responsible for the pathophysiological process is still uncharacterized. Autophagy is a fundamental cellular process for eukaryotes ongoing under various stress conditions. Therefore, we studied the role of autophagy in experiment colitis mouse model after Erbin deletion.

**Figure 5:**
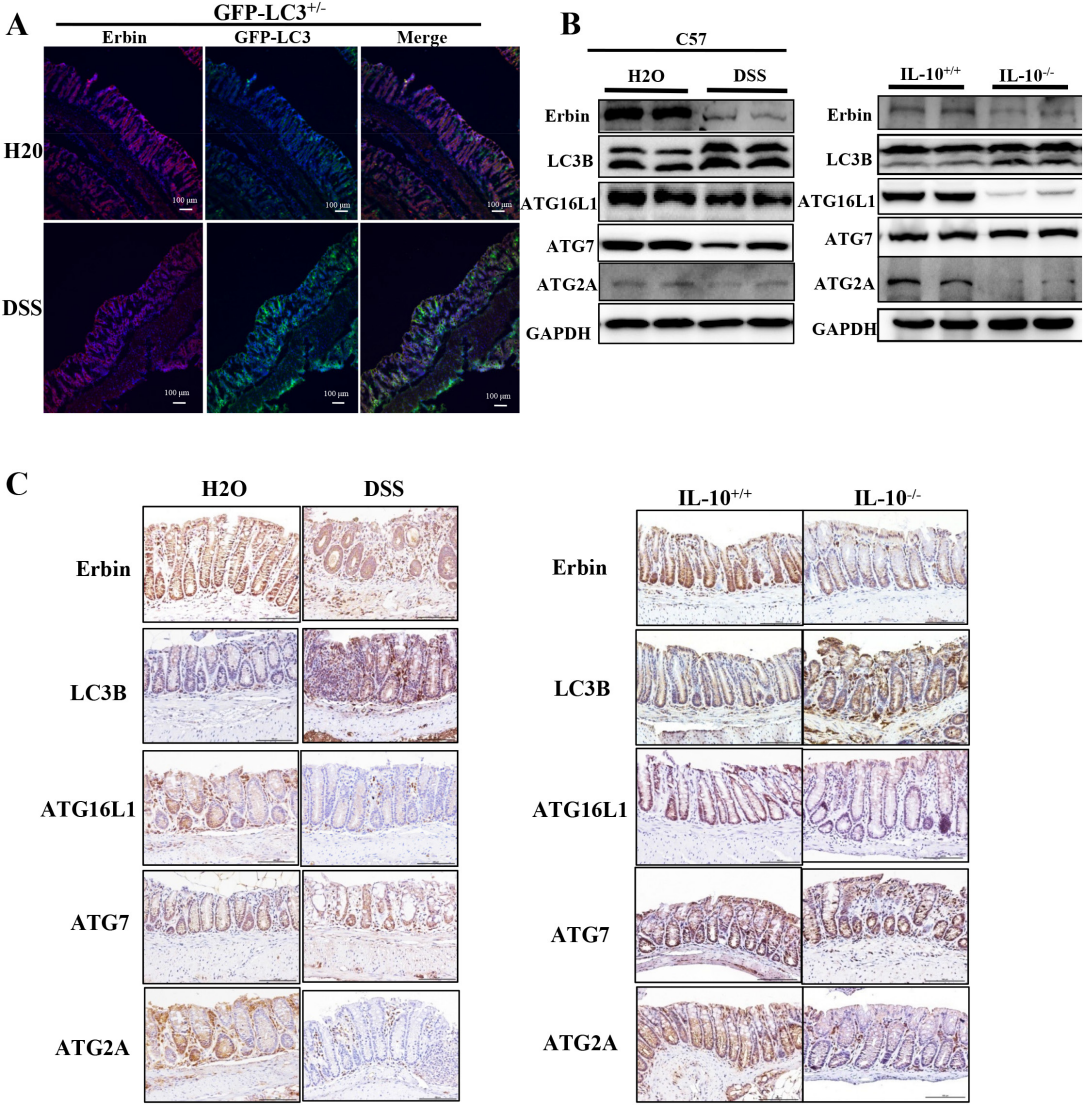
Expressions of Erbin, LC3, ATG16L1, ATG2A and ATG7 in DSS-induced experimental colitis mice and IL-10 knockout mice (IL-10^-/-^) **(A)** Expressions of Erbin (red) and LC3 (green) in colorectum of DSS -treated GFP-LC3^+/-^ mice by immunofluorescent staining and confocal microscopy. The nuclei were stained with DAPI (blue). **(B-C)** Expressions of Erbin, LC3, ATG16L1, ATG2A and ATG7 by Western Blots (WB) (B), and immunohistochemical staining (IHC) (C). Scale bars = 50 μm (A and C).

GFP-LC3^+/-^ mice (developed by Komatsu) [13], a kind of autophagy reporter mice, were used to observe the density and location of autophagic vacuoles *in vivo* after DSS treatment. Immunofluorescence of LC3 in isolated colon were examined using confocal microscopy. Upregulated LC3 expression accompanied with downregulated expression of Erbin was found in colonic epithelium of mice after DSS treatment (Figure 5A), suggesting the potential functional link between Erbin and autophagy program. Similarly, western blotting and immunohistochemical staining showed decreased expression of Erbin, increased expression of LC3B, decreased expressions of ATG16L1 and ATG2A in DSS-induced colitis mice and IL-10^-/-^ mice compared with that in control mice (Figure 5B and 5C).

**Figure 6:**
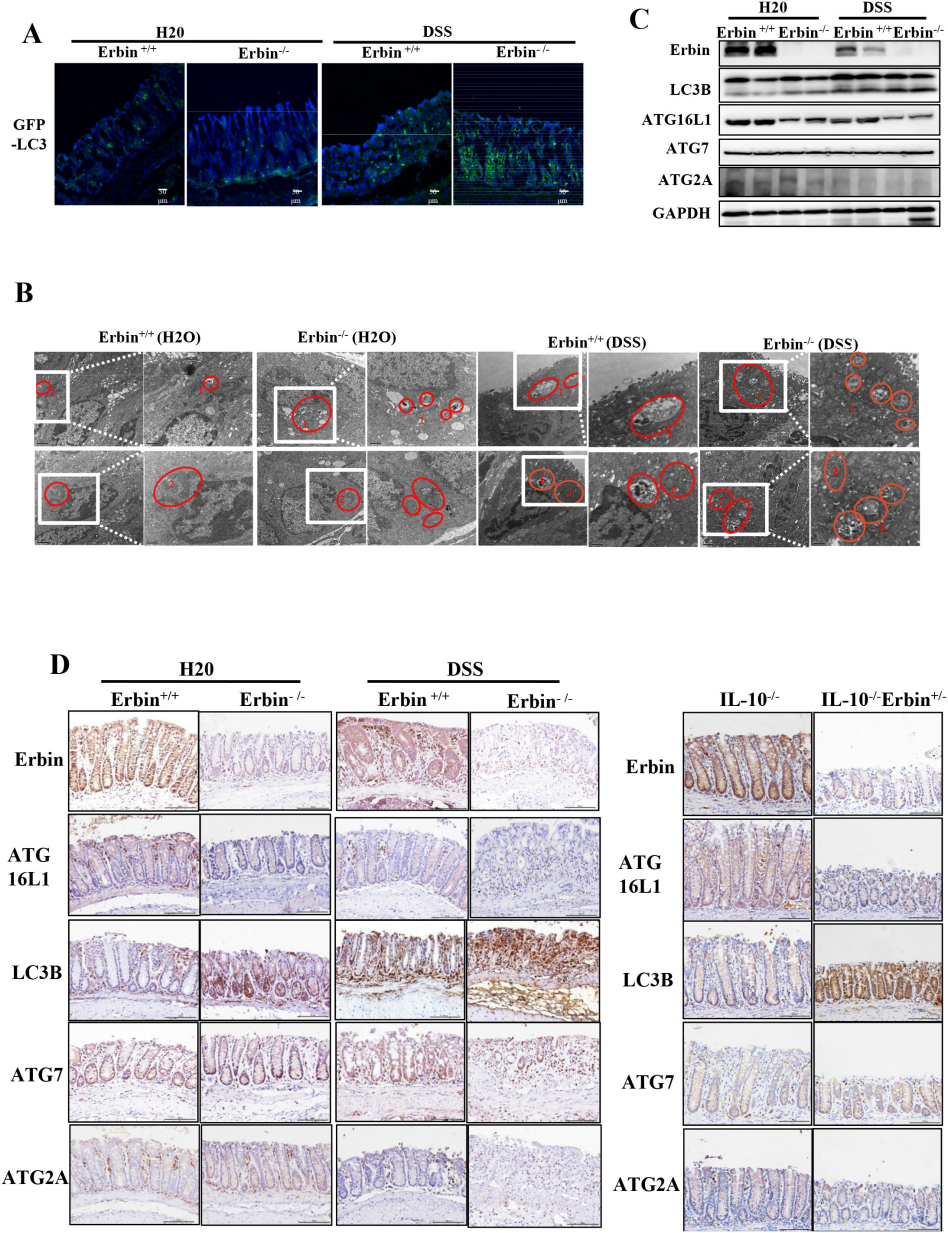
Autophagic program of colorectum in experiment colitis mouse model after Erbin was deleted **(A)** LC3 (green) in colorectum of DSS -treated GFP-LC3^+/-^ mice by immunofluorescent staining and confocal microscopy. The nuclei were stained with DAPI (blue). **(B)** Ultrastructural changes of autophagic/lysosomal vacuoles (labelled as 1 and mitochondrial (labelled as 2) in colorectal mucosa of Erbin^-/-^ mice treated with DSS. **(C-D)** Expression of Erbin, LC3, ATG16L1, ATG7, ATG2A of colorectum in DSS-induced wild type (WT) and Erbin^-/-^ mice by Western Blots (WB) (C), and immunohistochemical staining (IHC) in IL-10^-/-^ mice (D). Scale bar, 1 μm and 0.5 μm (B). Scale bar, 50 μm (A and D).

More importantly, GFP-LC3 puncta in epithelial cells within intestinal mucosa was greatly increased in DSS treated Erbin^-/-^ mice compared with that in WT control mice (Figure 6A). And, ratio of LC3-II/LC3-I expression was significantly upregulated in the Erbin^-/-^ mice as well as DSS-treated Erbin^-/-^ mice (vs Erbin WT mice or DSS-treated Erbin WT mice, respectively). Interestingly, the expressions of ATG16L1 but not ATG7 and ATG2A in Erbin^-/-^ mice with or without DSS treatment were lower than that in their corresponding WT mice (Figure 6A, 6C and 6D). Electron microscopy showed increased formation of autophagic/lysosomal vacuoles, accompanied with extensive mitochondrial swelling, in the colon of DSS treated Erbin^-/-^mice, suggesting the functional link between autophagy program and cell injury (Figure 6B).

### Knockdown of Erbin aggravates TNF-α inducing autophagic cell death *in vitro*

**Figure 7:**
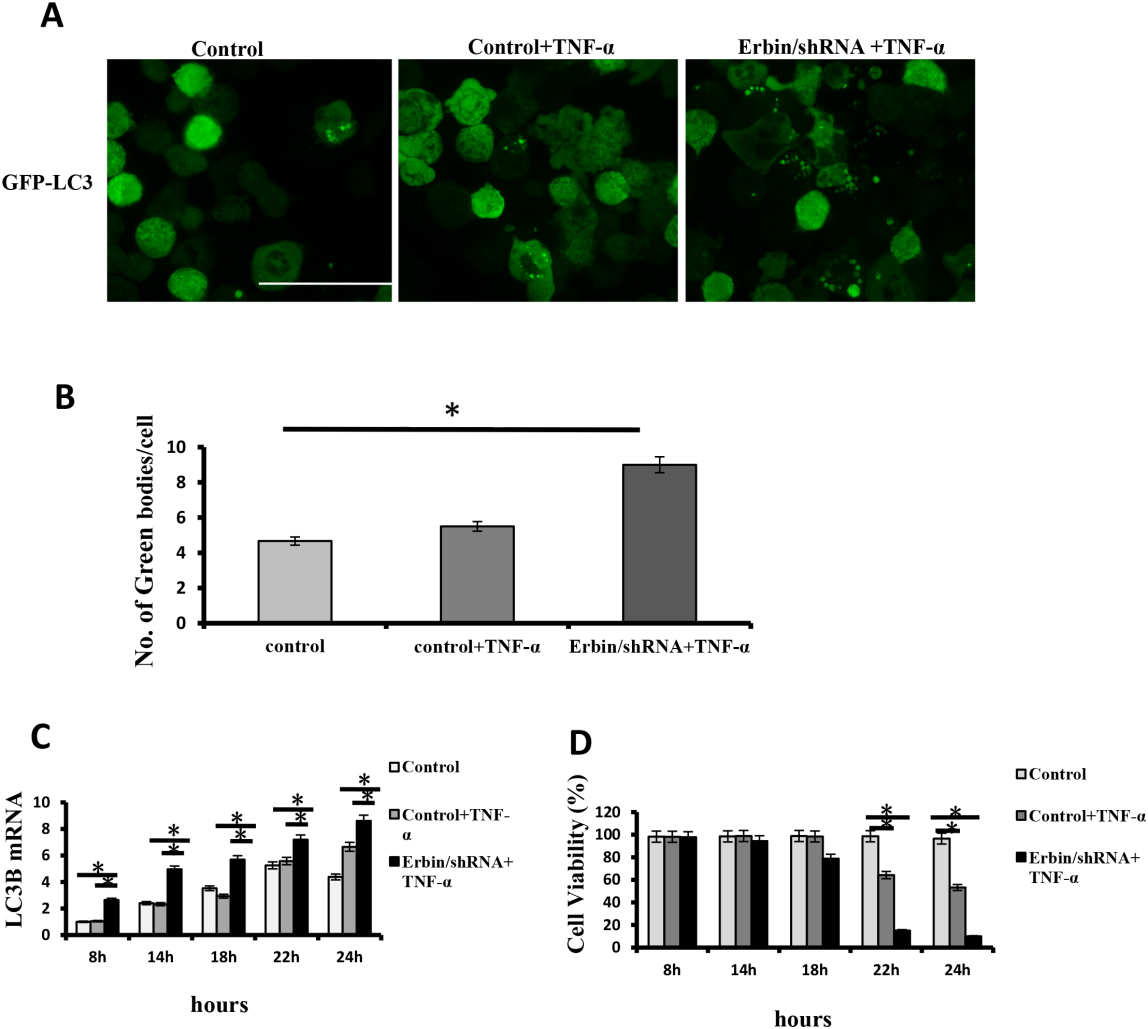
Autophagic cell death of CT26 cells after Erbin knockdown and TNF-α treatment **(A-B)** Immunofluorescent staining of LC3B (A) and the number of autophagic vacuoles per cell (B) in CT26 cells in presence of TNF-α after Erbin expression was knocked down by shRNA. **(C)** LC3B mRNA levels in CT26 cells after TNF-α treatment for 8, 14, 18, 22 and 24 hours by qPCR. **(D)** Cell viability (%) in CT26 cells after TNF-α treatment for 8, 14, 18, 22 and 24 hours by calculating the ratio of viable cells.

We found that Erbin deficiency exacerbated autophagy in experiment colitis mouse model *in vivo*. And, Erbin deletion led to damage of intestinal epithelium and excessive inflammatory response characterized as increased release of cytokine including IL-1β, TNF-α et al. TNF-α is critical for IBD development in humans and mice [14]. Therefore, we want to know whether Erbin deletion and TNF-α will synergistically contribute to autophagic cell death. qPCR analysis and immunofluorescence showed that LC3 expression in CT26 cells was significantly increased after TNF-α treatment. And, knockdown of Erbin and TNF-α synergistically promoted LC3 expression in CT26 cells (Figure 7A, 7B and 7C). Notably, knockdown of Erbin aggravates TNF-α inducing cell death *in vitro*. Flow cytometry showed that about 40% CT26 cells died 22 hours after TNF-α treatment. However, almost 90% CT26 cells died 22 hours after TNF-α treatment when Erbin expression was knocked down (Figure 7D).

### Inhibition of autophagy by Chloroquine attenuates excessive inflammatory response in the DSS-induced colitis mouse model of Erbin deletion

**Figure 8:**
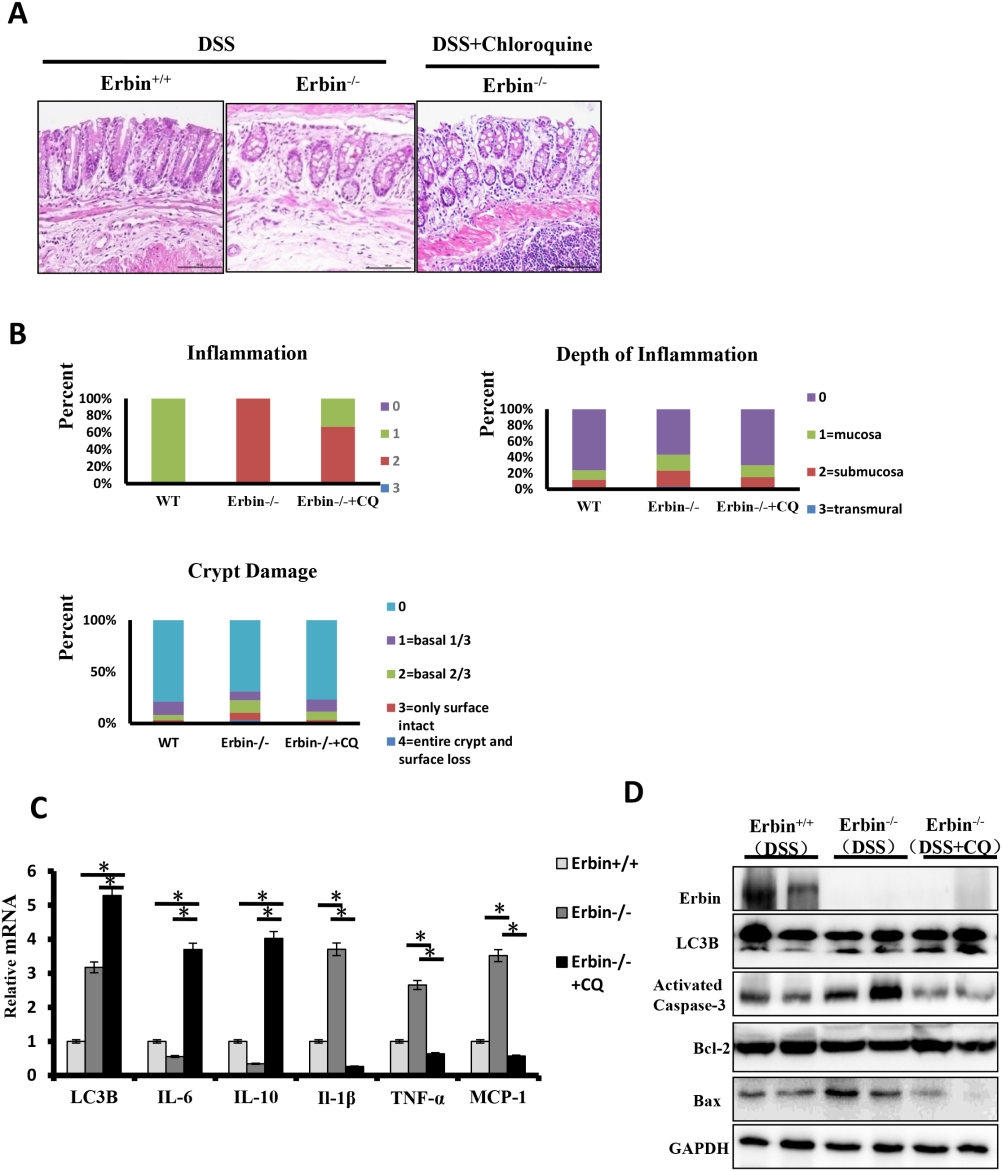
Intestinal inflammatory response and epithelial injury in the DSS-induced colitis mouse model of Erbin deletion after Chloroquine treatment **(A)** Representative H&E staining of colon samples acquired from DSS-induced wild type (WT) or Erbin^-/-^ mice and Chloroquine treated DSS-induced Erbin^-/-^ mice. **(B)** Histopathological scoring of the inflammation, depth of inflammation, and crypt damage of colon from mice indicated. **(C)** LC3B mRNA levels of Erbin, LC3B, IL-10, IL-6, IL-1β, TNF-a and MCP-1 in colon of samples from mice indicated by qPCR. **(D)** Expression of Erbin, LC3B, activated caspase-3, Bcl-2, and Bax in the colon from mice indicated by Western Blotting (WB).

We found that Erbin deletion and TNF-α synergistically contributed to autophagic cell death. Based on these results, we speculated that inhibition of the autophagy might attenuate cell death initiated by excessive autophagy program in the DSS-induced colitis mouse model of Erbin deletion. Chloroquine (CQ), a 4-aminoquinoline drug, which inhibits lysosomal acidification and therefore prevents autophagy by blocking autophagosome fusion and degradation, was used in our following experiments. Conversion of LC3-I to LC3-II was indicated in isolated colon of Erbin^-/-^ mice after CQ treatment (Figure 8C and 8D). HE staining results showed CQ treatment significantly attenuated inflammatory cell infiltration into mucosa and extensive damage of epithelium along with crypt destruction in DSS induced Erbin^-/-^ mice (Figure 8A and 8B). Functionally, expression pattern of cytokine was significantly rescued in DSS induced Erbin^-/-^mice after treated with CQ. Higher expressions of IL-1β, TNF-α and chemoattractant protein MCP-1 in DSS induced Erbin^-/-^ mice were inhibited and inhibitory expressions of IL-6 and IL-10 were upregulated after CQ treatment (Figure 8C). As is known to all, activation of caspase-3, Bax and Bcl-2 have been studied as biomarkers of cell death (apoptosis) [15, 16]. In accordance with above results, the expression of activated caspase-3 and the ratio of Bax/Bcl-2 could be fell in DSS induced Erbin^-/-^ mice after CQ treatment (Figure 8D).

## DISCUSSION

Erbin as a functional adaptor, is implicated in many key signal pathways. For a decade, Erbin was found to interact with Nod2 and inhibit Nod2-dependent NF-κB signaling and cytokine secretion [6]. Nevertheless, the role of Erbin in IBD is still unclear.

Here, we report that Erbin is a key IBD related gene. We found that Erbin is essential for intestinal homeostasis. Evolutionally, Erbin is conserved across species and highly expressed in mouse and even a model animal zebrafish. Physiologically, more severe intestinal inflammatory response and epithelial injury were found in 9-month old even 3.5-month old Erbin knockout mice. To further answer the role of Erbin in IBD, we used a line of experiment colitis mouse models. We found that Erbin knockout mice are more susceptible to DSS exposure, exhibiting more severe clinical symptoms such as loss of body weight, loose feces/watery diarrhea, fecal blood, intestinal inflammatory response and epithelial injury. More importantly, in samples from patients with ulcerative colitis, Erbin expression in the intestinal mucosa is significantly decreased. Until now, many IBD related genes have been reported based on many genetic or functional studies. GWAS (genome-wide association studies)-based studies have successfully identified NOD2, IL23R, and ATG16L1 as IBD related genes [6, 17–19]. Our previous study based on GWAS-based analysis exhibited that genetic variants of Nur77 gene linking to lower Nur77 expression was genetically related to IBD susceptibility [20]. Notably, in these IBD associated genes identified by genetic studies, NOD2 is an interacting partner of Erbin. Importantly, Nod2 is one member of important microbial sensors, which promotes pro-inflammatory signaling and cytokine networks to fight off bacterial infections. Mutations in Nod2 are associated with a number of human inflammatory disorders, including Crohn’s disease [21]. In zebrafish model [22], zebrafish with mutations in Nod2 have the highest disease-specific risk association for IBD.

**Figure 9:**
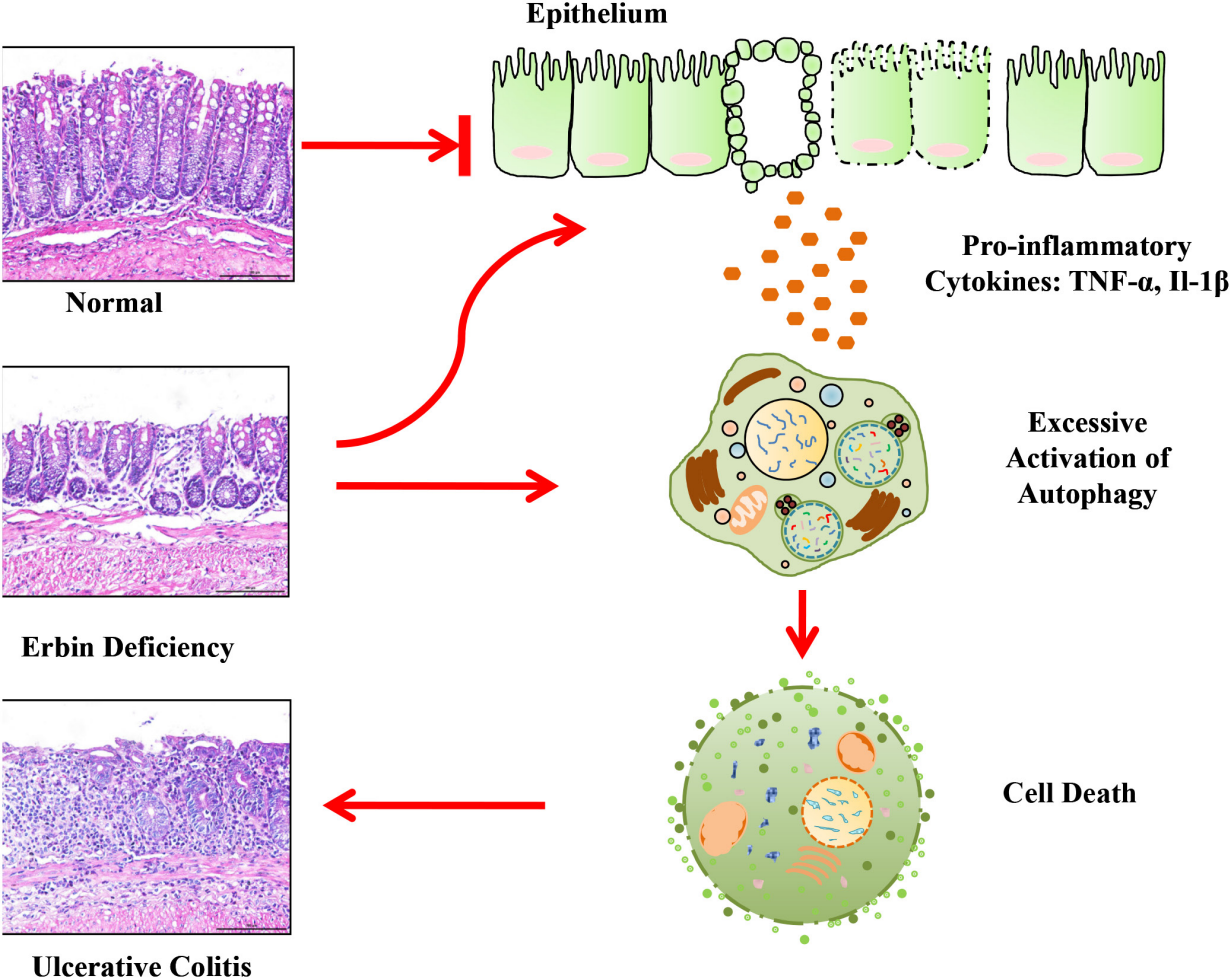
A proposed model for a novel role of Erbin in IBD and colonic homeostasis Erbin suppresses autophagic cell death and exerts a protective effect against inflammatory bowel disease.

Our study also clearly demonstrates that Erbin exerts a protective effect against inflammatory bowel disease by suppressing autophagic cell death. Autophagy, a fundamental cellular program involved in many physiologic or pathological conditions, is also required for intestinal homeostasis [23]. Several GWAS-based studies showed that mutations in multiple autophagy genes are highly linked to Crohn’s disease (CD) [24–26]. Moreover, defects of autophagy related genes will lead to enteritis. Intestinal conditional knockout of ATG16L1 resulted in increased intestinal inflammatory response and epithelial injury [26]. In accordance with this study, another study showed that ATG16L1-T300A risk allele show impaired clearance of patho-symbionts in ileal inflammation [27]. And, SNPs in ATG2A were found with risk for CD [28]. For a long time, many studies are focusing on defects of autophagy in chronic inflammation and IBD pathogenesis. It is traditionally accepted that down-regulation or defect of autophagy contribute to IBD pathogenesis. Logically, excessive activation of autophagy program is accompanied by not only the accumulation of autophagic vacuoles, but also an increase in autophagy that contributes to cell death. This interaction between dying cells and phagocytes reflects the baseline contribution of inflammation to normal tissue homeostasis [29]. Notably, here we found that autophagy activation is a characteristic linked with excessive intestinal inflammatory response and epithelial injury in a line of experiment colitis mouse model. More importantly, our results show that tumor necrosis factor can accelerate autophagic cell death owing to the deficiency of Erbin. Interestingly, a previous study showed high levels of autophagy is not only an indication for the cell to survive but also a potential signal for the cell to die [30]. Therefore, the deficiency in Erbin and excessive secreted cytokines, synergistically contributed to autophagic cell death, which in turn accelerated enteritis process. Inhibiting autophagy by Chloroquine could successfully attenuate the levels of the extent of inflammation, range of inflammation and crypt damage in Erbin^-/-^ mice. Based on these studies, we proposed a model for the role of Erbin in IBD and autophagy program (Figure 9). Briefly, within a certain range, increased autophagy program is a compensatory mechanism for the body to maintain gut homeostasis after Erbin is deleted. However, out of the compensatory limit, after excessive cytokine release, activation of autophagy program will transfer to autophagic cell death and accelerate the intestinal inflammatory response and epithelial injury.

Interestingly, we also uncover a key role of Erbin in regulation of Paneth cells. We found the number of Paneth cell in the terminal ileum of the Erbin^-/-^ mice was dramatically decreased. And, mRNA expression levels of Lysozyme (marker for Paneth cells) and Cryptidin were significantly decreased in ileum of the Erbin^-/-^ mice compared to that in WT mice with or even without DSS treatment. As we know, Paneth cells have an important function in maintaining the dynamic balance of enteric flora in health by producing a number of antibacterial products such as defensins, lysozyme, et al. Moreover, increasing evidence showed that Paneth cells are involved in intestinal inflammation. Defects in Paneth cells have been observed in high proportions of patients with CD, and are associated with a more aggressive CD phenotype. Impaired secretory functions of Paneth cells have been found described in patients with inflammatory bowel disease [31–33]. Thus, defect in Paneth cells will be another key factor contributing to excessive intestinal inflammatory response and epithelial injury due to Erbin deletion.

In summary, our study uncovers a novel role of Erbin in colonic homeostasis and IBD. Autophagic cell death or defect in Paneth cells are the main mechanisms responsible for intestinal epithelial injury after Erbin deletion.

## MATERIALS AND METHODS

### Erbin reporter mice, Erbin deletion or other transgenic mice

Erbin reporter mice donated by Dr Mei lin [9], were described previously [4], which express a beta-galactosidase enzyme, encoded by the lacZ gene. Erbin knockout (Erbin^-/-^) mice, in which exon 16 of Erbin gene was deleted by a CRISPR/Pro System, were established in our lab. IL-10^-/-^ mice were purchased from the Jackson Laboratory, and GFP-LC3^+/-^ was a kind gift from Dr Komatsu [13]. Mice were maintained following the Institutional Animal Care in a specific pathogen free environment of Soochow University. Offspring of above mice were genotyped using DNA isolated from the tail genomic DNA. Presence of the transgene was verified by PCR.

### X-gal staining

The mouse colon frozen blocks for section were prepared. *In Situ* β-galactosidase Staining Kit (Beyotime) was used. Tissues were perfused with ice-cold PBS followed by PBS containing 2% paraformaldehyde for 15 min. After fixation, samples were rinsed with PBS and stained with X-gal (Stratagene, Beyotime) overnight at 37°C in the dark. Stained specimens were rinsed with PBS and photographed under bright-field illumination using a Nikon Ti-S microscope.

### Whole -mount *in situ* hybridization in zebrafish

Albino zebrafish embryos were fixed in 4% PFA in phosphate-buffered saline (PBS) overnight at 4°C. Proteinase K treatment was done for 90 min. Whole -mount *in situ* hybridization with sense and antisense RNA probes was performed. The Erbin probes were labeled with digoxigenin (DIG) using the DIG 30 end labeling kit (Roche). To decrease background in fish, hybridized fish were washed in PBST overnight at 4°C after anti-digoxigenin incubation. Erbin expression was visible after approximately 2 hr of staining at room temperature. Fish were fixed in 4% paraformaldehyde after staining and photographed in glycerol. Image acquisition Embryos, larvae were analyzed with Leica MZFLIII microscopes and subsequently photographed with digital cameras.

### DSS-induced mouse model of colitis

All mice experiments were performed with male mice of 8 to 10 weeks of age unless noted otherwise. We induce colitis by DSS. Mice received standard chow diet during the experiment. Control mice received untreated water ad libitum and the colitis group received indicated amounts of 2.5% DSS (Sigma) dissolved in water for 7 days.

To test the effect of Chloroquine on enteritis, Erbin^+/+^ and Erbin^-/-^ mice received 1.5% DSS for 5 days, another group Erbin^-/-^ mice received 1.5% DSS, meanwhile injected intraperitoneally with Chloroquine (CQ; Sigma-Aldrich, St. Louis, MO, USA) dissolved in phosphate-buffered saline (PBS) once a day (60 mg/kg of body weight) for 5 days.

### Measurement of colitis severity and histological scoring

After day 7, the colitis severity was estimated by measuring body weight loss, colon length and colon weight. Paraffin-embedded sections were stained for H&E. Histology was scored by a pathologist in a blinded fashion as a combination of inflammatory (score 0-3), depth of inflammation (score 0-3) and crypt damage (score 0-4) according to Cooper et al [34]. The crypt damage was scored as followed: grade 0, intact crypt; grade 1, loss of the basal one-third of crypt; grade 2, loss of the basal two-thirds of the crypt; grade 3, loss of entire crypt with the surface epithelium remaining intact; grade 4, loss of both the entire crypt and surface epithelium. These changes were described as the percentage involvement by the disease process: (1) 1 to 25%; (2) 26 to 50%; (3) 51 to 75%; and (4) 76 to 100%. Sores for inflammation were subjectively graded 0-3 and their extent of involvement was estimated at: (1) 1 to 25%; (2) 26 to 50%; (3) 51 to 75%; an (4) 76 to 100% of surface area.

### Immunohistochemistry

All immunohistochemistry was performed on formalin-fixed, paraffin-embedded samples. Sections were de-waxed and rehydrated through a graded series of ethanol. Endogenous peroxidase was quenched by incubation in hydrogen peroxide (3% w/v in methanol for 15 minutes). Antigen retrieval was performed by boiling in a pressure cooker for 10 minutes in either citrate buffer. Sections were blocked for 30 minutes with 10% normal goat serum (Life Technologies), prior to incubation with primary antibody overnight at 4°C. Section and slides were stained for Erbin (A303-762A; Bethyl), LC3B (3868; CST), ATG16L1 (8089; CST), ATG7 (BA3527-2; Boster), ATG2A (15011; CST), respectively.

### Western blotting

Colon tissues or cells were washed with Hanks' balanced salt solution. After washing, the tissue or cells was homogenized in a lysis buffer and a tablet of protease inhibitor cocktail (Roche Diagnostics, Mannheim, Germany). The extracts of the tissue were separated on a 8% or 12% denaturing polyacrylamide gel and transferred to a polyvinylidene difluoride membrane by electroblotting. Transferred membranes were then blocked for 1 h at room temperature with 5% non-fat dried milk in 1× Tris-buffered saline and 0.1% Tween-20 before incubating with antibodies against Erbin (A303-762A; Bethyl), LC3B (3868; CST), ATG16L1 (8089; CST), ATG7 (8558; CST), ATG2A (15011; CST), Bax (bsm-33279; Bioss), Bcl-2 (bsm-33411; Bioss), and active Caspase3 (bsm-33199; Bioss) respectively. After washing with Tris-buffered saline and 0.1% Tween-20, the membranes were incubated with a peroxidase-conjugated secondary antibody, diluted at 1:10000 in blocking buffer, for 1 hour at room temperature. Detection was performed by incubating the membranes with enhanced chemiluminescent (ECL) substrate (Pierce) to visualize the protein bands and scanned blots with the ChemiDoc XRS gel documentation system (Bio-rad).

### Real-time RT-PCR

Portions of colons were isolated and thoroughly washed with saline. cDNA was synthesized from total RNA isolated from colon and analyzed by real-time RT-PCR. For mRNA, cDNA was generated from 1 μg total RNA per sample using the Transcriptor First Strand cDNA Synthesis Kit (Roche). qPCR was performed by using the ABI StepOnePlus and the SYBR® Select Master Mix (ABI). mRNA expression was normalized using detection of 18S Ribosomal RNA. Results are represented as fold induction using the ΔΔCt method with the control set to 1.

### Cell culture and reagents

Mouse CT26 cell line was purchased from ATCC. Cells were cultured in RPMI 1640 with 10% (v/v) fetal bovine serum and were maintained at 37 °C in 5% CO2 incubator. TNF-α (Genescript) was used at a final concentration of 100 ng/ml. The lentivirus vector down-expressing Erbin was prepared for CT26 cells using shRNA targeting Erbin or a negative control vector according to the manufacturer’s protocol. shRNA lentiviral particles were treated in six-well plates in the presence of polybrene (6 μg/ml). Cells were treated with puromycin (2 μg/ml) to generate stable Erbin knockdown clones. Erbin siRNAs and negative control siRNAs were purchased from RiboBio. The constructs GFP-LC3, pCMV-Myc-Erbin were generated by PCR and confirmed by sequencing. CT26 cells were transfected with plasmids or siRNAs using Lipofectamine 2000 (Invitrogen) according to the manufacturer’s instruction. All transfections were performed in triplicate for each experiment.

### Immunofluorescence

The mouse colon frozen blocks for section were prepared. Then, frozen blocks embedded in Optimal Cutting Temperature compound were sectioned at 10 μm using Leica CM1950. Cells were permeabilized with 0.3% Triton X-100 for 10 minutes followed by fixation with 2-4% Methanol for 15 minutes, and blocked with 3% sheep serum at room temperature for 60 minutes. Then, probed with primary antibodies anti-LC3B and anti-Erbin described before overnight at room temperature, stained with anti-Rabbit IgG H&L (abcam #ab6717) for 1 hour at room temperature. Nuclei were visualized by staining with DAPI (Sigma, USA) for 2 minutes. The stained cells were observed with inverted fluorescence microscope (Nikon Ni-U). Autophagy was measured by quantitation of GFP-LC3 puncta per cell using fluorescence microscopy. All GFP-LC3 puncta quantitation was performed by an observer blinded to experimental condition.

### Transmission electron microscopy

After the 2.5% DSS for 3 days, Erbin^-/-^ and WT mice (n = 3 for each group) were sacrificed, and their colons were harvested after the perfusion with heparinized PBS and the fixation with 4% (w/v) paraformaldehyde in 0.1 M phosphate buffer solution (pH 7.4). The colons were cut in small cubes (roughly 0.5-mm sides) as samples for transmission electron microscopy (TEM). The cut tissue samples were washed three times for 5 minutes each in 0.1 M phosphate buffer solution (pH 7.4) containing 4% sucrose. The samples were then post fixed by 1% (w/v) OsO4 and 1% (w/v) potassium ferrocyanide in 0.1 M phosphate buffer solution (pH 7.4) for one hour at room temperature in a draft chamber, and washed three times for one minute in PBS. All the samples were dehydrated in a graded series of ethanol solutions: 50%, 70%, and 90% (v/v) ethanol on ice, then twice in 100% ethanol for 10 minutes at room temperature. Then the sample tissues were incubated in 50% (v/v) epoxy resin mixture dissolved in 100% ethanol for 60 minutes at room temperature. The dehydrated tissues were infiltrated with resin mixture for one hour at room temperature. This process was then repeated. The embedding mixtures were polymerized in the cavities of silicon rubber for 2 days at 60°C. Marked regions of the resulting resin blocks were cut into ultra-thin sections of 60-90 nm. The thin sections were stained with 2% (w/v) uranyl acetate solution for one hour and briefly washed three times in distilled water. The sections then were incubated in lead-staining solution for two minutes and washed briefly three times in distilled water, then scanned by an electron microscope (JEM 1200EX; JEOL).

### Paneth cell staining

Paneth cells were visualized by phloxine-tartrazine staining. After 7 μm sections were deparaffinized and rehydrated, the nuclei were stained with hematoxylin for 3 minutes. After a brief wash in water, the sections were stained in the phloxine solution 0.5% (w/v) phloxine B and 0.5% (w/v) calcium chloride in water for 10 minutes, successively rinsed with water and Cellosolve (2-ethoxy ethanol, Sigma-Aldrich Co., St Louis, MO), and immersed in a saturated tartrazine solution for 5 minutes. After dehydration, the sections were cleared and enclosed for microscopy. The results are expressed as the percentage of the Paneth cells per crypt.

### The annexin V assay

For cell death assay, 1×10^5^ treated cells were incubated with Annexin V/propidium iodide for 15 minutes at room temperature in the dark. Briefly, cells were plated and treated with the reagents. Subsequently, the cells were washed twice with PBS and resuspended in 1 mL of 1×binding buffer. Cells undergoing early apoptosis were analyzed by counting the early-stage apoptotic cells that stained positive for Annexin V-FITC and negative for PI, and dead cells including late-stage apoptotic cells and necrotic cells as Annexin V-FITC and PI positive using Beckman Coulter FC500 flow cytometer. Each test was repeated in triplicate.

### Statistical analysis

Data were analysed using a Student's t-test (with 95% CI) or Newman-Keuls post-test following one-way analysis of variance where multiple groups are compared. Data are expressed as means±SEM. p Values less than 0.05 were considered significant.

## SUPPLEMENTARY MATERIALS FIGURES


