# Supplementary material for: Erbin exerts a protective effect against inflammatory bowel disease by suppressing autophagic cell death
Source: Oncotarget. 2018 Jan 4;9(15):12035–49. doi: 10.18632/oncotarget.23925 (PMC5844727; doi:10.18632/oncotarget.23925)
Supplement: Supplementary file 1 [file oncotarget-09-12035-s001.pdf]

Erbin exerts a protective effect against inflammatory bowel disease by suppressing autophagic cell death

SUPPLEMENTARY MATERIALS

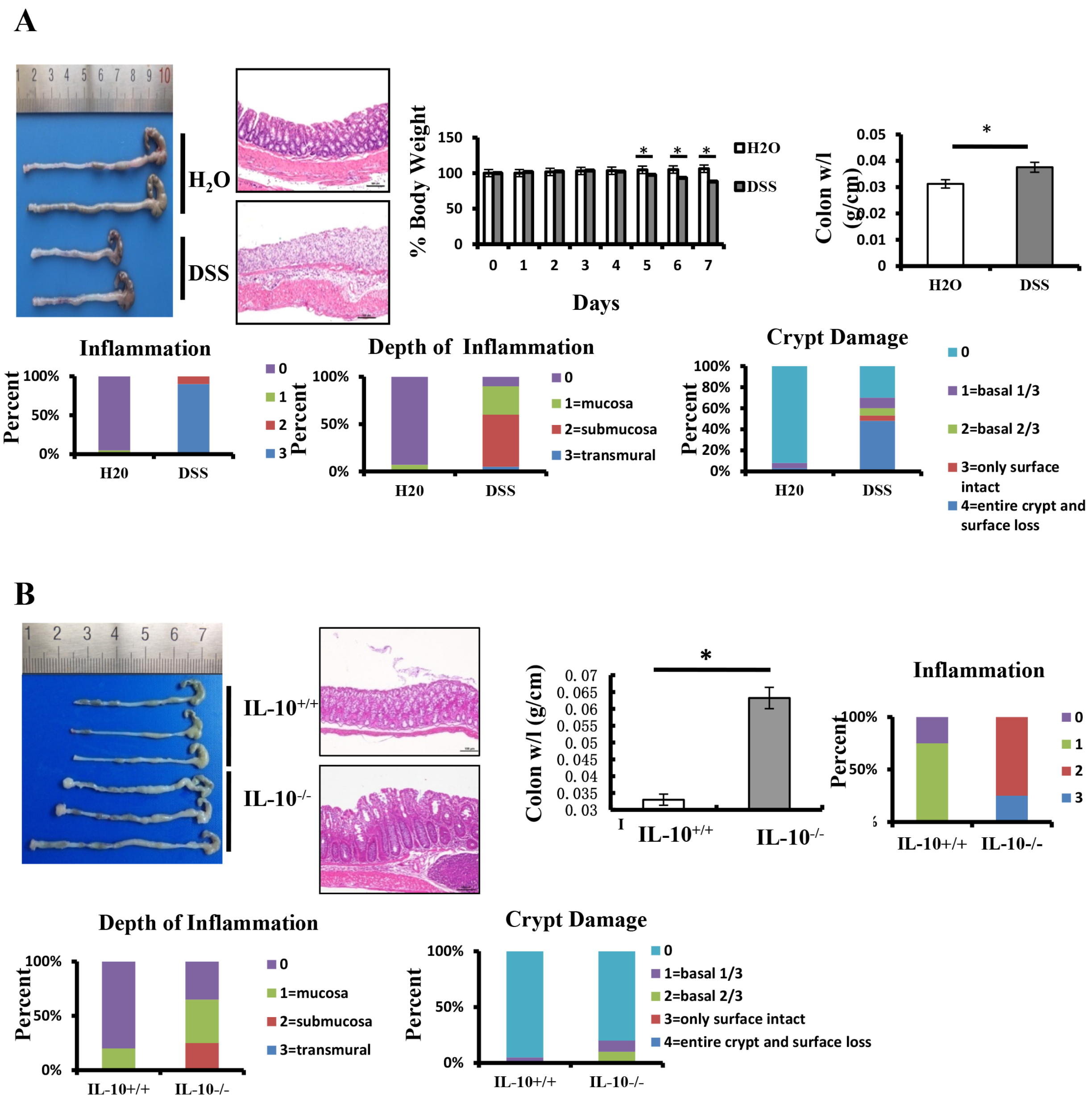

Supplementary Figure 1: Two types of experimental colitis mouse models: DSS- induced colitis mouse model and IL-10 deletion (IL-10$^{-/-}$) mouse model. (A) Gross appearance, body weight during 7 days, the ratio of colon weight and length, histological scorings for intestinal inflammatory response and epithelial injury in mice after DSS treatment. **(B)** Gross appearance, the ratio of colon weight and length, histological scorings for intestinal inflammatory response and epithelial injury in IL-10$^{-/-}$ and IL-10$^{+/+}$ mice. Scale bar, 50 µm (A and B). Data are expressed as means±SEM. $^{*}P < 0.05$ (t-test) (A and B).

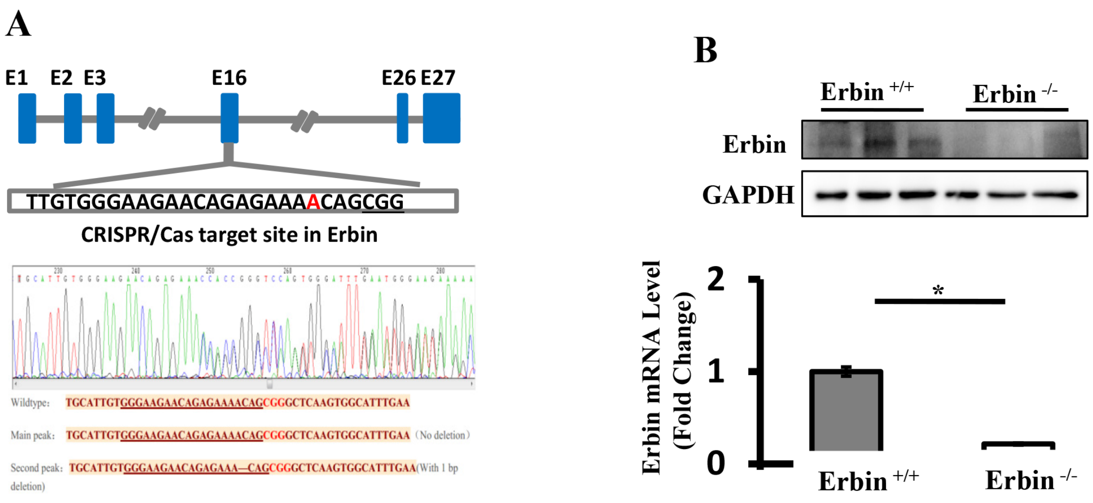

Supplementary Figure 2: Generation of Erbin deletion (Erbin$^{-/-}$) mice. (A) Schematic diagram of Erbin deletion by CRISPR/Pro systems. **(B)** Expression of Erbin by Western Blots (WB) and RT-PCR in Wild type (WT) and Erbin$^{-/-}$ mice. Data are expressed as means±SEM. $^{*}P < 0.05$ (t-test) (B).

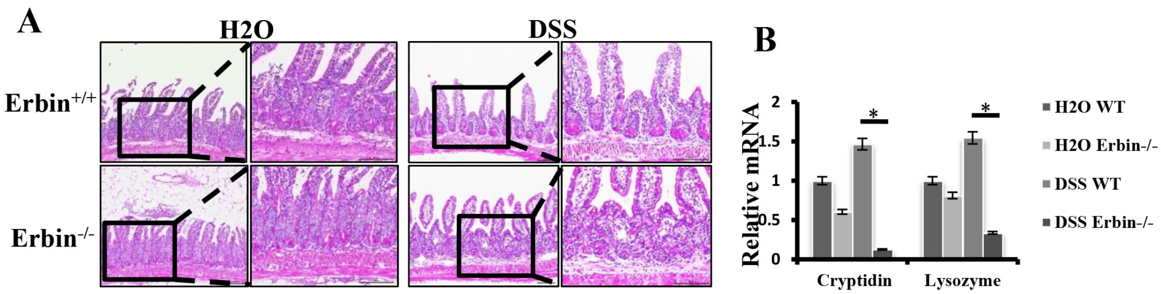

Supplementary Figure 3: Paneth cells in Erbin deletion mice. **(A)** phloxine-tartrazine staining for Paneth cell in small intestine of Wild type (WT) and Erbin$^{-/-}$ mice with or without DSS treatment. **(B)** mRNA expression of Cryptidini and Lysozyme by RT-PCR from terminal ileum of WT and Erbin$^{-/-}$ mice with or without DSS treatment. Data are expressed as means±SEM. Scale bars = 50 µm (A). $^{*}P < 0.05$ (t-test) (B).
